# The influence of encoding strategy on associative memory consolidation across wake and sleep

**DOI:** 10.1101/lm.053765.123

**Published:** 2023-09

**Authors:** Dan Denis, Ryan Bottary, Tony J. Cunningham, Mario-Cyriac Tcheukado, Jessica D. Payne

**Affiliations:** 1 Department of Psychology, University of York, York YO10 5DD, United Kingdom; 2 Institute for Graduate Clinical Psychology, Widener University, Chester, Pennsylvania 19013, USA; 3 Center for Sleep and Cognition, Psychiatry Department, Beth Israel Deaconess Medical Center, Boston, Massachusetts 02215, USA; 4 Division of Sleep Medicine, Harvard Medical School, Boston, Massachusetts 02215, USA; 5 Department of Psychology, University of Notre Dame, Notre Dame, Indiana 46556, USA

## Abstract

Sleep benefits memory consolidation. However, factors present at initial encoding may moderate this effect. Here, we examined the role that encoding strategy plays in subsequent memory consolidation during sleep. Eighty-nine participants encoded pairs of words using two different strategies. Each participant encoded half of the word pairs using an integrative visualization technique, where the two items were imagined in an integrated scene. The other half were encoded nonintegratively, with each word pair item visualized separately. Memory was tested before and after a period of nocturnal sleep (*N* = 47) or daytime wake (*N* = 42) via cued recall tests. Immediate memory performance was significantly better for word pairs encoded using the integrative strategy compared with the nonintegrative strategy (*P* < 0.001). When looking at the change in recall across the delay, there was significantly less forgetting of integrated word pairs across a night of sleep compared with a day spent awake (*P* < 0.001), with no significant difference in the nonintegrated pairs (*P* = 0.19). This finding was driven by more forgetting of integrated compared with not-integrated pairs across the wake delay (*P* < 0.001), whereas forgetting was equivalent across the sleep delay (*P* = 0.26). Together, these results show that the strategy engaged in during encoding impacts both the immediate retention of memories and their subsequent consolidation across sleep and wake intervals.

The ability to form new associations between previously unrelated pieces of information is a critical function of human learning and memory. Our ability to form such associations is highly dependent on the type of strategy we use when learning. One particularly powerful technique is forming mental visualizations that conceptually integrate new items together in a coherent scene (Graf and Schacter 1985, 1989; Diana et al. 2008; Murray and Kensinger 2012; Parks and Yonelinas 2015). For example, if trying to remember the word pair duck–car, one might imagine a duck driving a car, a duck sleeping on top of the roof of a car, or a car in the shape of a duck. In other words, integrative visualization involves binding together discrete pieces of information into a single mental representation. The mnemonic benefit of this strategy is that during recollection one can better bring to mind both of the items simultaneously. The formation of new associative memory is believed to be facilitated by the hippocampus, along with other medial temporal lobe structures such as the perirhinal and parahippocampal cortices in the case of integrative memory visualization (Gold et al. 2006; Staresina and Davachi 2006, 2010; Eichenbaum et al. 2007; Suzuki 2007). Reactivation of these structures, especially the hippocampus, during sleep is believed to contribute to the process of memory consolidation (Peigneux et al. 2004; Sterpenich et al. 2021).

To be remembered in the long term, memories need to undergo a period of consolidation, whereby newly acquired memories are stabilized and made more resistant to forgetting (Rasch and Born 2013; Klinzing et al. 2019). Sleep has been suggested to be an optimal time for memory consolidation to occur, with a wealth of evidence supporting the view that memory is superior after a night of sleep compared with an equivalent period of time spent awake (Berres and Erdfelder 2021; Hokett et al. 2021). In particular, sleep appears to facilitate the strengthening of new associative memories (Studte et al. 2015; Schreiner et al. 2021) as well as integrate new memories into existing knowledge structures (Ellenbogen et al. 2007; Alger and Payne 2016). While the benefit of sleep on associative declarative memory is generally thought to be through making initially recalled memories more resistant to forgetting (Fenn and Hambrick 2013; Denis et al. 2020; Muehlroth et al. 2020), there is evidence that sleep can help to make initially forgotten memories more accessible (Dumay 2016).

Certain boundary conditions appear to moderate the benefit of sleep on the consolidation of memories (Berres and Erdfelder 2021; Cordi and Rasch 2021). These include the amount of information to be encoded (Feld et al. 2016; Kolibius et al. 2021), the number of exposures to each stimulus (Drosopoulos et al. 2007; Schapiro et al. 2017; Denis et al. 2020), and the degree of prior relatedness between items (Payne et al. 2012b; Havas et al. 2018). In a previous experiment, we found that sleep benefitted memory consolidation when participants were able to successfully visualize an integrated scene that bound the two word pair objects together, but not when participants failed to visualize a scene (Denis et al. 2020). One interpretation of this finding is that an initial binding between items, supported by integrative visualization, is essential in order for sleep to consolidate that new association. This is supported by evidence that the amount of hippocampal engagement during encoding predicts subsequent consolidation during sleep (Rauchs et al. 2011). However, a limitation of our prior study was that we did not directly compare integrative versus nonintegrative encoding strategies. It is thus unclear whether sleep preferentially benefits associative memories based on their level of integration at encoding or whether visualization itself, irrespective of integration, creates a deep enough memory trace that sleep can act on.

In the present study, we sought to directly compare the effect of sleep on memory consolidation for new associative memories encoded with either an integrative or nonintegrative visualization strategy (cf., Murray and Kensinger 2012, 2014). We hypothesized that an initial binding between new item associations, facilitated by an integrated encoding strategy, would lead to enhanced memory performance compared with memories formed with a nonintegrative strategy and augmented sleep-associated memory consolidation compared with a wake-filled delay.

## Results

**Figure 1.**
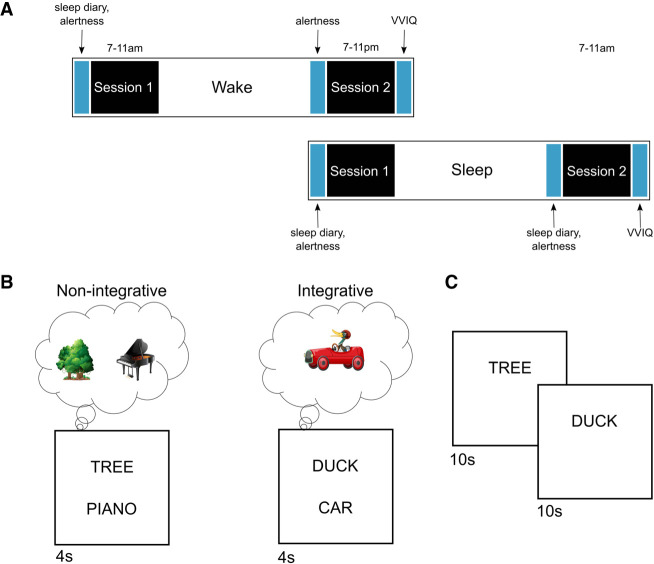
Experimental design. (*A*) Study timeline. During session 1, participants performed encoding followed by an immediate cued recall test. During session 2, participants performed a second cued recall test. (VVIQ) Vividness of Visual Imagery Questionnaire. (*B*) Illustration of the encoding task. During the nonintegrative block, participants were instructed to generate separate mental images for the two items in as much vivid detail as possible and not to imagine the items together or interacting with each other in any way. During the integrative block, participants were told to generate a mental image that combined the two items into a single visual representation in as much vivid detail as possible. (*C*) During the cued recall tasks, participants were shown the first word in the pair and were required to recall the second word. (Images in *B* use assets from Freepik.com.)

Participants were randomly assigned to either a sleep (*n* = 47) or wake (*n* = 42) delay group. The groups followed the exact same protocol, which consisted of two experimental sessions spaced ∼12 h apart (Fig. 1A). Sleep participants performed session 1 in the evening and session 2 the following morning. Wake participants performed session 1 in the morning and session 2 in the evening. During session 1, participants encoded 80 word pairs using either a nonintegrative (*n* = 40) or integrative (*n* = 40) encoding strategy (Fig. 1B; see the Materials and Methods for details). Half of the word pairs consisted of high-imageability words, and the other half consisted of low-imageability words. Due to floor effects for the low-imageability pairs (<10% accuracy in the nonintegrative condition) (see the Materials and Methods), analyses were only performed on the high-imageability items. For the nonintegrative encoding block, participants were instructed to “generate separate mental images for each item in as much vivid detail as possible and to not imagine the items interacting together in any way.” For the integrative block, participants were instructed to “generate a single mental image that combined both word pair items into a single mental representation in as much vivid detail as possible.” After encoding all of the pairs, participants then performed an immediate cued recall test of all word pairs (Fig. 1C). In session 2, participants performed a second cued recall test of all word pairs. Due to COVID-19 restrictions, the entire protocol took place online.

The average time between sessions 1 and 2 was 714 min (SD = 121 min). There was a significantly longer delay between sessions in the sleep group (*M* = 795 min, SD = 90 min) compared with the wake group (*M* = 625 min, SD = 80 min; *t*_(60.97)_ = 7.94, *P* < 0.001). Within the sleep group, participants reported sleeping an average of 472 min (SD = 75 min) between sessions 1 and 2. The sleep and wake groups did not differ in terms of demographics, sleep duration in the three nights prior to the study, diurnal preference, subjective alertness at either experimental session, or trait imagery vividness (all *P*s > 0.07) (Supplemental Table S1).

### Visualization during encoding

Participants reported being highly successful at performing the visualization task. There was a trending main effect of encoding condition on visualization success, with participants slightly more successful at visualizing in the nonintegrative condition (*M* = 0.97, SD = 0.05) compared with the integrative condition (*M* = 0.95, SD = 0.06), but it failed to reach significance (*F*_(1,86.51)_ = 3.84, *P* = 0.053). In terms of the vividness of visualization, there was no difference between the integrative (*M* = 3.45, SD = 0.43) and nonintegrative (*M* = 3.43, SD = 0.46) encoding strategies (*F*_(1,87)_ = 0.73, *P* = 0.40). There were no main effects or interactions involving the delay group (all *P* > 0.08).

### Memory performance

**Figure 2.**
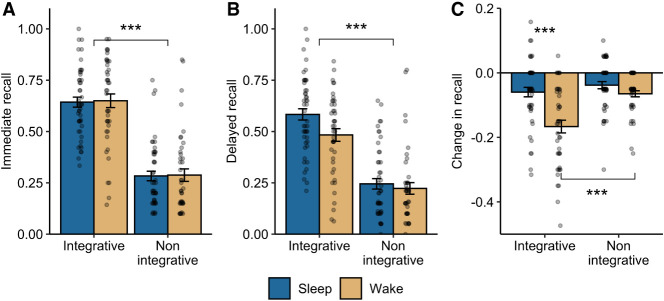
Effects of encoding strategy and delay on memory. (*A*) Immediate recall accuracy. *Y*-axis indicates the proportion of correctly recalled word pairs. (*B*) Delayed recall accuracy. (*C*) Change in recall between session 1 and session 2 (delayed − immediate recall). Error bars represent the between-subjects standard error. (***) *P* < 0.001.

We next examined memory performance at the immediate test (see Table 1 for all memory scores). We observed a significant main effect of encoding condition (*F*_(1,87)_ = 212.14, *P* < 0.001), with vastly superior recall for word pairs encoded with the integrative strategy (*M* = 0.65, SD = 0.19) compared with the nonintegrative strategy (*M* = 0.29, SD = 0.178) (Fig. 2A). There was no main effect of group (*F*_(1,87)_ = 0.03, *P* = 0.86) or a significant encoding condition × delay group interaction (*F*_(1,87)_ = 0.002, *P* = 0.96). At the delayed test (Fig. 2B), we again observed a significant main effect of encoding condition (*F*_(1,87)_ = 166.93, *P* < 0.001; integrative *M* = 0.54, SD = 0.20; nonintegrative *M* = 0.23, SD = 0.18). We also observed a trend toward a main effect of group (*F*_(1,87)_ = 3.68, *P* = 0.058), with numerically higher recall accuracy in the sleep delay group (*M* = 0.41, SD = 0.25) compared with the wake group (*M* = 0.35, SD = 0.23). The interaction was not significant (*F*_(1,87)_ = 2.83, *P* = 0.096).

**Table 1.**
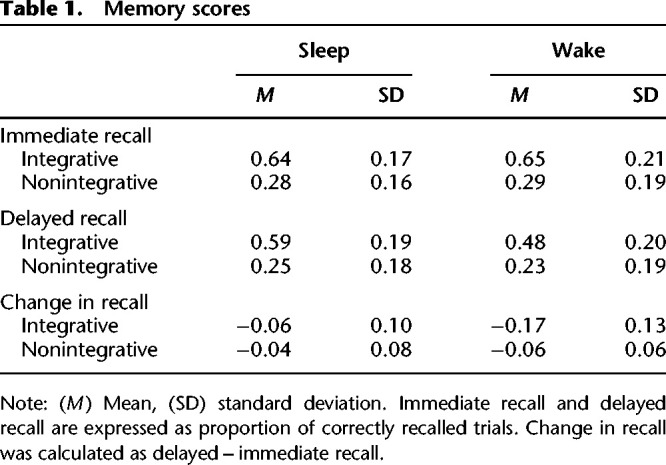
Memory scores

We next examined the change in recall between the two tests (Fig. 2C). There was a significant main effect of group (*F*_(1,87)_ = 20.72, *P* < 0.001), with reduced forgetting after sleep (*M* = −0.05, SD = 0.09) compared with wake (*M* = −0.12, SD = 0.11). This effect was superseded by a significant interaction between encoding strategy and delay group (*F*_(1,87)_ = 8.56, *P* = 0.004), demonstrating that the beneficial effect of sleep on memory varied by the strategy engaged in during encoding. For word pairs encoded with an integrative strategy, there was less forgetting after sleep (*M* = −0.06, SD = 0.10) compared with wake (*M* = −0.17, SD = 0.123; *P* < 0.001). For nonintegrative word pairs, there was no difference in forgetting between the groups (sleep: *M* = −0.04, SD = 0.08; wake: *M* = −0.07, SD = 0.06; *P* = 0.19). This effect was driven by increased forgetting of integrated word pairs in the wake condition compared with nonintegrative pairs (*t*_(87)_ = 5.11, *P* < 0.001). Within the sleep group, retention of the integrated and nonintegrated pairs was equivalent (*t*_(87)_ = 1.14, *P* = 0.26).

Across encoding conditions and delay groups, change in recall was correlated with immediate memory accuracy (*r* = −0.36, *P* < 0.001). Therefore, we reran our analyses using an adjusted change score, regressing out immediate recall accuracy (see the Materials and Methods; Antony et al. 2018a). Using these adjusted scores, the same pattern of results emerged (significant encoding condition × delay group interaction; *F*_(1,87)_ = 9.89, *P* = 0.002).

We excluded 16 participants based on evidence that they did not follow task instructions during at least one session (e.g., no key presses were recorded on any experimental trial) (see the Materials and Methods); however, we note that primary results were unchanged when those participants were included (Supplemental Table S2).

These results suggest that across a 12-h delay, there is less forgetting of integrated word pairs when the delay is filled with sleep compared with wake. To complement these findings, we ran a trial-level mixed-effects model in which we included test session (immediate or delayed) as an additional fixed-effects factor, trial as an additional random effect, and visualization success and imageability rating as continuous covariates. This result matched the trial-averaged approach, with a significant three-way interaction between encoding strategy, delay group, and test session (*P* = 0.010). Similarly, follow-up tests revealed a significant difference between wake and sleep for integrated word pairs at the delayed test only (*P* = 0.04).

### Item fate

We next conducted an item fate analysis to examine the trajectory of word pairs across the delay period. Specifically, we examined whether the effect of sleep was primarily driven by higher maintenance of initially recalled word pairs or by increased gains of initially forgotten pairs (Dumay 2016). There were significantly more maintained (*M* = 0.76, SD = 0.19) than gained (*M* = 0.20, SD = 0.18; *t*_(61)_ = 18.76, *P* < 0.001) pairs overall. Using normalized proportions (see the Materials and Methods), we then examined how item fate differed between encoding conditions and groups. This analysis revealed a significant main effect of item fate (*F*_(1,207.70)_ = 9.31, *P* = 0.003), with a higher rate of maintained (*M* = 0.99, SD = 0.25) relative to gained (*M* = 0.90, SD = 0.79) items. This main effect was qualified by an interaction between item fate and encoding condition (*F*_(1,203.16)_ = 33.18, *P* < 0.001). The rate of gains made was significantly higher for integrated (*M* = 1.21, SD = 0.87) compared with nonintegrated (*M* = 0.46, SD = 0.43; *t*_(204.31)_ = 6.725, *P* < 0.001) word pairs, whereas item maintenance was equivalent across both encoding conditions (*t*_(150.42)_ = 0.17, *P* = 0.87). There was also a main effect of group (*F*_(1,125.58)_ = 4.54, *P* = 0.035), with the sleep group showing both higher maintenance (*M* = 1.06, SD = 0.24) and higher gains (*M* = 0.98, SD = 0.90) compared with the wake group (maintained: *M* = 0.93, SD = 0.24; gained: *M* = 0.77, SD = 0.57). There was no interaction between item fate and delay group (*F*_(1,207.7)_ < 0.001, *P* = 0.99), and the three-way interaction was not significant (*F*_(1,203.16)_ = 1.70, *P* = 0.19).

### Correlational analyses

Within the sleep group, total self-reported sleep time between encoding and recall was not correlated with change in recall for either integrated (*r* = 0.11, *P* = 0.54) or nonintegrated (*r* = 0.03, *P* = 0.86) word pairs. Similarly, the total delay length between experimental sessions did not correlate with change in recall in either the sleep or the wake group (all *r*s < 0.27, all *P*s > 0.16). Trait-level vividness of visual imagery did not correlate with either immediate memory accuracy or the change in memory (all *r*s < 0.12, all *P*s > 0.35). Finally, the sleep and wake groups did not differ in terms of sleep duration in the three nights prior to the study, diurnal preference, subjective alertness at either experimental session, or trait imagery vividness (all *P*s > 0.07) (Supplemental Table S1).

## Discussion

This study set out to test the hypothesis that associative memories formed using an integrative encoding strategy would be better consolidated over a night of sleep compared with memories encoded with a nonintegrative strategy. In support of our hypothesis, we found a significant interaction between encoding condition and delay group, such that a significant benefit of sleep was seen for the integrated word pairs only. However, the driver of this effect was unexpected. Rather than preferential consolidation of integrated items within the sleep group, we instead found increased forgetting of integrated items within the wake group, compared with the nonintegrated items. We discuss our findings in more detail below.

First, and in accordance with previous research, we found superior memory for the integrated word pairs, as evidenced at both the immediate and delayed tests (Murray and Kensinger 2012, 2014). As such, it appears that integrated visualization promotes initially stronger and more durable associative memory representations than nonintegrative encoding. Importantly, these results were seen when only successfully visualized trials were included, and the vividness of the visualization did not differ between the two conditions. As such, it appears to be the technique of integration itself, rather than visualization success or vividness, driving the heightened recollection of integrated word pairs.

We expected sleep to consolidate integrated word pairs to a greater degree than nonintegrated word pairs, on the basis that integrated visualization would create an initial binding between items that could be strengthened during sleep. Instead, within the sleep group, consolidation of integrated and nonintegrated was equivalent. In our previous report, we found that visualization success moderated the sleep benefit (Denis et al. 2020). Given that we only included successfully visualized trials in the present study, it may be the case that the act of successfully visualizing is what is important for a sleep benefit to be seen, as opposed to the specific type of visualization strategy used. Visualization success was at ceiling in the present study, meaning that we were unable to compare between successfully and unsuccessfully visualized trials.

During wake, we observed significantly more forgetting of integrated memories compared with nonintegrated memories. As such, the sleep–wake difference in integrated memory consolidation can be better described as a cost of wake, rather than a benefit of sleep (Payne et al. 2012b). This finding was unexpected, given that prior studies have found that more weakly encoded information (as assessed by initial memory performance) is forgotten to the greatest extent over a wake delay (Drosopoulos et al. 2007; Denis et al. 2020, 2021). Because far more integrated word pairs were initially remembered, this led to an overall greater amount of material to retain over the delay period. Whereas most of this information was retained in the sleep group, there was significant forgetting in the wake group. This could be interpreted in terms of sleep increasing the amount of information that can be retained across 12 h. Integrative encoding facilitates easier recall in a cued recall paradigm because when presented with one word of the pair, it should be easier to bring to mind the associate, given they were encoded in the same internal visualization (Murray and Kensinger 2012). As such, recall of nonintegrated word pairs likely had a higher retrieval threshold to surpass in order to be remembered. A second possibility then is that initially recalled nonintegrative items had a high underlying memory strength and so decayed more slowly over the wake delay. This leaves open the possibility that different results would have been obtained using a different type of recall test, such as free recall. We used a cued recall procedure here, given our specific interest in the associative memory (Studte et al. 2015). However, future studies using different kinds of memory tests would help drive this area of research forward.

Memory for all items was tested both before and after the delay period. This is consistent with many other studies of sleep and memory (Plihal and Born 1997; Schabus et al. 2004) and with empirical and meta-analytic evidence suggesting that sleep benefits are more reliably observed when a presleep test is included (Schoch et al. 2017; Lipinska et al. 2019). Nonetheless, it is not possible to completely disentangle sleep and retrieval practice effects in this design. Other studies have found that the presence of a presleep retrieval test eliminates subsequent sleep effects (Bäuml et al. 2014; Antony and Paller 2018; Abel et al. 2019), potentially through raising the memory strength of successfully retrieved memories beyond a level sleep cannot benefit further (Bäuml et al. 2014). The fact that we saw a sleep effect in the current study could suggest that memories were of a low enough initial strength that successful presleep retrieval raised memory strength to within a range that sleep could still benefit.

Consistent with prior work using this task, the order of encoding conditions was not counterbalanced, with nonintegrative encoding always occurring first (Murray and Kensinger 2012, 2014). This was motivated by prior research that found that participants were unable to successfully perform nonintegrative visualization after having first performed integrative visualization (Murray and Kensinger 2012). This study also reported that when two blocks of integrative word pairs were encoded, there were no memory differences between the first and second block (Murray and Kensinger 2012). This argues in favor of our findings primarily reflecting differences in encoding strategy rather than condition order. Nevertheless, it remains possible that the effects of encoding strategy at the immediate test could be at least partly driven by recency effects, which then carried over to the second session. A fruitful avenue for future research would be to repeat this experiment in a between-subjects design, where participants use either integrative or nonintegrative visualization during encoding.

We used the “classic” 12-h sleep versus wake design, motivated on the basis that this design elicits sleep effects in an online setting, giving us increased confidence that the effect of sleep could be successfully studied here (Ashton and Cairney 2021; Ashton et al. 2022; Denis et al. 2022). However, this design has two limitations that should be addressed in future research. First, wake participants were exposed to more interfering information throughout the delay, meaning we cannot rule out a passive protection from interference role of sleep from this data set alone. However, we point to other studies showing that a sleep benefit still exists even when the amount of waking interference is equivalent between groups (Gais et al. 2006; Talamini et al. 2008; Payne et al. 2012a; Denis et al. 2020). Second, the sleep and wake groups trained and tested at different times of day, leaving open the possibility that circadian or time of day differences could account for our findings. We think this is unlikely, however, given other research showing sleep effects in a daytime nap paradigm, where training and test times are the same between groups (Mednick et al. 2003; Alger et al. 2010; Denis et al. 2021; Farhadian et al. 2021). We also note that there were no group differences in subjective alertness ratings between groups, ruling out differences in vigilance as a possible factor. An inherent limitation of an online experimental setting is reduced experimental control. This likely explains the higher than expected exclusion rate due to participants not following the protocol and also may explain the overall longer delay length in the sleep group. Although this is a limitation, the latter issue serves to further exhibit the powerful effect sleep has on memory, given that significant sleep effects were observed in spite of an overall longer delay interval.

The study of salience cues such as emotion as prioritization cues for sleep-associated memory has received a great deal of attention in recent years (Kim and Payne 2020; Davidson et al. 2021; Cunningham et al. 2022; Denis et al. 2022). Other factors that may moderate sleep-associated memory consolidation, such as encoding strategy, are relatively less well understood. Here, we showed that compared with wake, memory for word pairs encoded with an integrative visualization strategy was better after sleep compared with a nonintegrative strategy. Future research should work to further unpack the boundary conditions of memory consolidation during sleep.

## Materials and Methods

### Participants

Participants were 125 University of Notre Dame undergraduate students. While no a priori power analysis to determine sample size was performed, group sizes were more than double that of our previous report on this topic (Denis et al. 2020). Of those, 34 participants’ data were excluded due to technical errors (*n* = 6 reported some technical error such as not being able to load the experiment or the experiment crashing) or a failure to adhere to the experimental procedure (*n* = 16 did not make a single keyboard response during at least one session, and *n* = 12 did not return for the second session). Two additional participants were removed due to being statistical outliers on the memory consolidation measure (>1.5 times the interquartile range). Therefore, a final sample of 89 participants was included in the analysis (*M*_age_ = 19 yr, [SD = 1.1 yr], 67% female) (see Supplemental Table S1 for full demographics). Inclusion criteria were aged 18–30 yr old and free of any current psychiatric, neurological, or sleep-related disorders. Participants were recruited through Sona (Sona Systems, https://www.sona-systems.com). All participants provided informed consent prior to participation and were compensated for their time with course credit. All procedures were approved by the University of Notre Dame Institutional Review Board.

### Materials

Stimuli were 160 concrete nouns (mean length of five letters) obtained from the Medical Research Council psycholinguistic database (Wilson 1988). Half of the word pairs were highly imageable (*M* = 632, SD = 7, theoretical range 100–700), and the other half were of low imageability (*M* = 475, SD = 19). This was done to test an additional hypothesis regarding how the nature of encoding materials may influence consolidation. However, due to floor effects at the immediate test (<10% accuracy in the nonintegrative condition) (Supplemental Table S3), it was not possible to reliably assess consolidation of the low-imageability trials. Therefore, analyses focused solely on the high-imageability word pairs. An additional 20 words (10 pairs) were used as practice trials.

All participants, regardless of condition, completed a sleep diary of bed and rise times in the three nights prior to participating in the experiment. Sleep participants completed an additional sleep diary for the night between experimental sessions. Diurnal preference was assessed via a single item from the Morningness–Eveningness Questionnaire: “One hears about ‘morning’ and ‘evening’ types of people. Which ONE of these types do you consider yourself to be?” (Horne and Ostberg 1976). Before the beginning of each experimental session, participants completed the Stanford sleepiness scale to assess subjective alertness (Hoddes et al. 1972). At the end of the experiment, the trait vividness of visual imagery for all participants was measured with the Vividness of Visual Imagery Questionnaire (Marks 1973).

### Procedure

Due to COVID-19 restrictions, the entire protocol took place online. After completing an initial screening survey, eligible participants were invited to participate in the main study, which consisted of two experimental sessions placed ∼12 h apart. Participants were instructed to abstain from alcohol in the 24 h prior to the experiment and abstain from both alcohol and caffeine on experimental days. Eligible participants were randomly allocated to either a sleep (*n* = 47) or wake (*n* = 42) delay group. Participants in the sleep group were instructed to complete experimental session 1 in the evening between 7:00 and 11:00 p.m. and session 2 the following morning between 7:00 and 11:00 a.m. Participants in the wake group were told to complete session 1 in the morning (between 7:00 and 11:00 a.m.) and session 2 that same evening (7:00–11:00 p.m.). Participants were told not to nap in between experimental sessions. Session 1 consisted of an encoding task followed by an immediate cued recall test of all word pairs. Session 2 consisted of a second, delayed cued recall test of all pairs. The encoding and recall tasks were programmed using jsPsych (de Leeuw 2015) and hosted on the Cognition.run platform (https://www.cognition.run).

#### Encoding

Participants studied 80 word pairs across two blocks of 40 trials each. Each block consisted of 20 high-imageability and 20 low-imageability word pairs. In the first block, participants first practiced the nonintegrative visualization strategy (five trials) before beginning the nonintegrative block. After completing the nonintegrative portion, they next practiced the integrative visualization strategy (five trials) before performing the integrative encoding strategy. Imagery strategy was not counterbalanced, as prior work using this task has demonstrated that participants are unable to successfully perform nonintegrative visualization after learning integrative visualization (Murray and Kensinger 2012). Participants were instructed on the encoding strategies as follows.

For the nonintegrative encoding block, participants were instructed to “generate separate mental images for each item in as much vivid detail as possible and to not imagine the items interacting together in any way” (Fig. 1A). Each word pair was displayed on the screen one at a time for 4 sec. Following each word pair, participants rated how successful they were at generating two separate images on a scale from 1 to 4, where 1 = “I could only imagine the items together” or “no image generated for one or both items,” 2 = “I could imagine the items separately but with little detail,” 3 = “I could imagine each item separately and in some detail,” and 4 = “I could imagine each item separately and in very vivid detail.”

For the integrative encoding block, participants were instructed to “generate a single mental image that combined both word pair items into a single mental representation in as much vivid detail as possible” (Fig. 1A). Again, each word pair appeared for 4 sec, after which participants rated their success at integrative visualization on a 1–4 scale where 1 = “I could only imagine the items separately” or “no image generated for one or both items,” 2 = “I could imagine the items together but with little detail,” 3 = “I could imagine each item together and in some detail,” and 4 = “I could imagine each item together and in very vivid detail.”

#### Cued recall

Participants took part in two identical cued recall tests. The first one occurred immediately following encoding, and the second occurred 12 h later (Fig. 1B). On each trial, the first word of a word pair studied during encoding appeared on the screen, and participants were instructed to type in the second word of the pair. The word appeared on the screen for 10 sec and then advanced automatically if no keyboard input was detected.

### Memory analysis

For all analyses of memory, only word pairs for which participants were able to successfully form a visualization were included (provided either a 3 or 4 response when rating visualization success). Because we were interested in the effect of visualization strategy, we reasoned that we should only analyze trials in which participants reported being able to successfully implement the strategy. Memory at each recall test was calculated as the proportion of correct trials that participants were able to visualize. When scoring responses, obvious spelling errors (coded by two independent scorers; discrepancies resolved via consensus) and pluralizations were considered correct.

Memory consolidation was quantified as the change in recall between the two tests (i.e., delayed − immediate recall performance). To remove any influence of immediate memory performance on the change in recall, we also calculated an adjusted change score following a previously used method (Antony et al. 2018a,b). For this, we regressed the change in recall against immediate recall performance (Supplemental Fig. S1A) and obtained the residual. Next, we added back the mean change in recall (across all participants, groups, and encoding conditions) to produce an adjusted change score (Supplemental Fig. S1B). Primary results did not differ when using the unadjusted or adjusted scores, so we report the unadjusted data here.

To examine the recall trajectory between the immediate and delayed recall tests, we performed an item fate analysis following established procedures (Dumay 2016). Maintained items were calculated as the number of word pairs recalled at both immediate and delayed test, divided by the number of word pairs recalled at the immediate test (i.e., the maximum possible number of maintained items). Gained word pairs were calculated as the number of items recalled at the delayed test that were not recalled at the immediate test, divided by the total number of word pairs minus the number of items recalled at the immediate test (i.e., the maximum possible number of gained items). Because gaining a pair was expected to be far less frequent than maintaining an item in memory (Dumay 2016; Denis et al. 2020), we normalized the proportion of maintained and gained pairs for the purpose of testing for an interaction. Specifically, the proportion of maintained and gained items was divided by their respective means, computed across groups and encoding conditions (Dumay 2016).

### Statistical analysis

All analyses were performed using R (https://www.R-project.org). The effects of encoding strategy condition (integrative and nonintegrative) and delay group (sleep and wake) on visualization success and memory were assessed using a series of linear mixed-effects models implemented in the lme4 package (Bates et al. 2015). Encoding strategy, delay group, and their interaction were entered as fixed effects, and participant was entered as a random effect as follows:

*dv* ∼ encoding_strategy × delay_group + (1 participant), where *dv* was either proportion of trials successfully visualized, visualization vividness rating, immediate recall accuracy, delayed recall accuracy, or the change in recall. For the item fate analysis, the same procedure was used except item fate (maintained or gained) and their interactions were entered as additional fixed effects. Statistical significance was established via Satterthwaite's degrees of freedom approximation method implemented in the lmerTest package (Kuznetsova et al. 2017). Follow-up pairwise contrasts were carried out as appropriate using the emmeans package (https://CRAN.R-project.org/package=emmeans). We complemented these analyses with a trial-level mixed-effects approach, which included test session (immediate or delayed) as an additional fixed factor and trial as an additional random effect. We also included visualization success and imageability ratings for each trial as additional covariates. All correlational analyses (e.g., correlations between sleep time and change in recall) were performed using Pearson's correlation implemented in the rstatix package (https://CRAN.R-project.org/package=rstatix).

## Supplementary Material

Supplement 1
