# Supplementary material for: The influence of encoding strategy on associative memory consolidation across wake and sleep
Source: Learn Mem. 2023 Sep;30(9):185–91. doi: 10.1101/lm.053765.123 (PMC10547373; doi:10.1101/lm.053765.123)
Supplement: Supplement 1 [file Supplemental_material.docx]

**Table S1**. Sample demographics and questionnaire assessments

|  | Sleep | | Wake | | stat | sig |
| --- | --- | --- | --- | --- | --- | --- |
|  | M / % | SD | M / % | SD |  |  |
| Age (years) | 19 | 1.2 | 19 | 1.02 | 1.08 | .28 |
| Sex |  |  |  |  |  |  |
| Female | 65 |  | 69 |  | 0.02 | .88 |
| Male | 35 |  | 31 |  |  |  |
| Ethnicity |  |  |  |  |  |  |
| Hispanic or Latino | 22 |  | 13 |  | 0.37 | .54 |
| Not Hispanic or Latino | 78 |  | 87 |  |  |  |
| Race |  |  |  |  |  |  |
| American Indian or Alaska Native | 0 |  | 0 |  | 3.74 | .44 |
| Asian | 13 |  | 6 |  |  |  |
| Black or African American | 3 |  | 10 |  |  |  |
| Native Hawaiian or Pacific Islander | 3 |  | 0 |  |  |  |
| White | 78 |  | 74 |  |  |  |
| Not listed | 3 |  | 10 |  |  |  |
| Diurnal preference^1^ | 2.85 | 0.87 | 2.58 | 0.92 | 1.19 | .24 |
| Evening preference | 67% |  | 61% |  | 0.04 | .85 |
| Preference preference | 33% |  | 39% |  |  |  |
| Session 1 subjective alertness^2^ | 2.60 | 1.04 | 2.60 | 0.99 | 0.002 | .99 |
| Session 2 subjective alertness | 2.85 | 1.12 | 2.37 | 1.43 | 1.75 | .084 |
| Vividness of visual imagery^3^ | 56.74 | 12.03 | 59.55 | 8.95 | 1.15 | .25 |
| Baseline nights^4^ |  |  |  |  |  |  |
| Bed time | 00:42 | 01:02 | 00:30 | 01:08 | 0.64 | .53 |
| Rise time | 08:46 | 01:07 | 08:38 | 01:05 | 0.23 | .82 |
| Total sleep time (mins) | 483.13 | 44.91 | 487.74 | 60.00 | 0.50 | .62 |
| Consolidation night^5^ |  |  |  |  |  |  |
| Bed time | 00:45 | 01:24 |  |  |  |  |
| Rise time | 08:37 | 11:05 |  |  |  |  |
| Total sleep time (mins) | 471.91 | 74.79 |  |  |  |  |

*Note*. Sex, Ethnicity, and Race expressed as percentage of the sample and group differences were assessed by chi-square. All other variables shown as the mean and standard deviation, with group differences assessed by t-tests. ^1^ A larger number indicates a greater evening preference (theoretical range 1 - 4). Evening preference = % of participants responding 3 or 4 to diurnal preference question. Morning preference = % of participants responding 1 or 2 to diurnal preference question. ^2^ Subjective alertness assessed by the Stanford sleepiness scale. A larger number indicates feeling more sleepy/less alert (theoretical range 1 - 7). ^3^ A larger number indicates more vivid visual imagery (theoretical range 16 - 80). ^4^ Baseline nights refer to the average of the three nights prior to starting the experiment. ^5^ Consolidation night refers to the night between session 1 and session 2 in the sleep group.

**Table S2**. Memory scores including all participants

|  | Sleep | | Wake | |
| --- | --- | --- | --- | --- |
|  | M | SD | M | SD |
| **Immediate recall** |  |  |  |  |
| Integrative | .58 | .24 | .59 | .24 |
| Non-integrative | .22 | .18 | .22 | .20 |
| **Delayed recall** |  |  |  |  |
| Integrative | .53 | .23 | .44 | .21 |
| Non-integrative | .20 | .18 | .17 | .18 |
| **Change in recall** |  |  |  |  |
| Integrative | -.05 | .12 | -.16 | .13 |
| Non-integrative | -.01 | 10 | -.04 | -.06 |

*Note.* M = Mean, SD = Standard deviation. Immediate and delayed recall expressed as proportion of correctly recalled trials. Change in recall calculated as delayed - immediate recall.

**Table S3**. Memory scores for low imageability trials

|  | Sleep | | Wake | |
| --- | --- | --- | --- | --- |
|  | M | SD | M | SD |
| **Immediate recall** |  |  |  |  |
| Integrative | .45 | .24 | .46 | .24 |
| Non-integrative | .06 | .13 | .09 | .15 |
| **Delayed recall** |  |  |  |  |
| Integrative | .41 | .23 | .31 | .20 |
| Non-integrative | .05 | .13 | .07 | .15 |
| **Change in recall** |  |  |  |  |
| Integrative | -.04 | .15 | -.15 | .16 |
| Non-integrative | -.01 | .06 | -.02 | .08 |

*Note.* M = Mean, SD = Standard deviation. Immediate and delayed recall expressed as proportion of correctly recalled trials. Change in recall calculated as delayed - immediate recall.


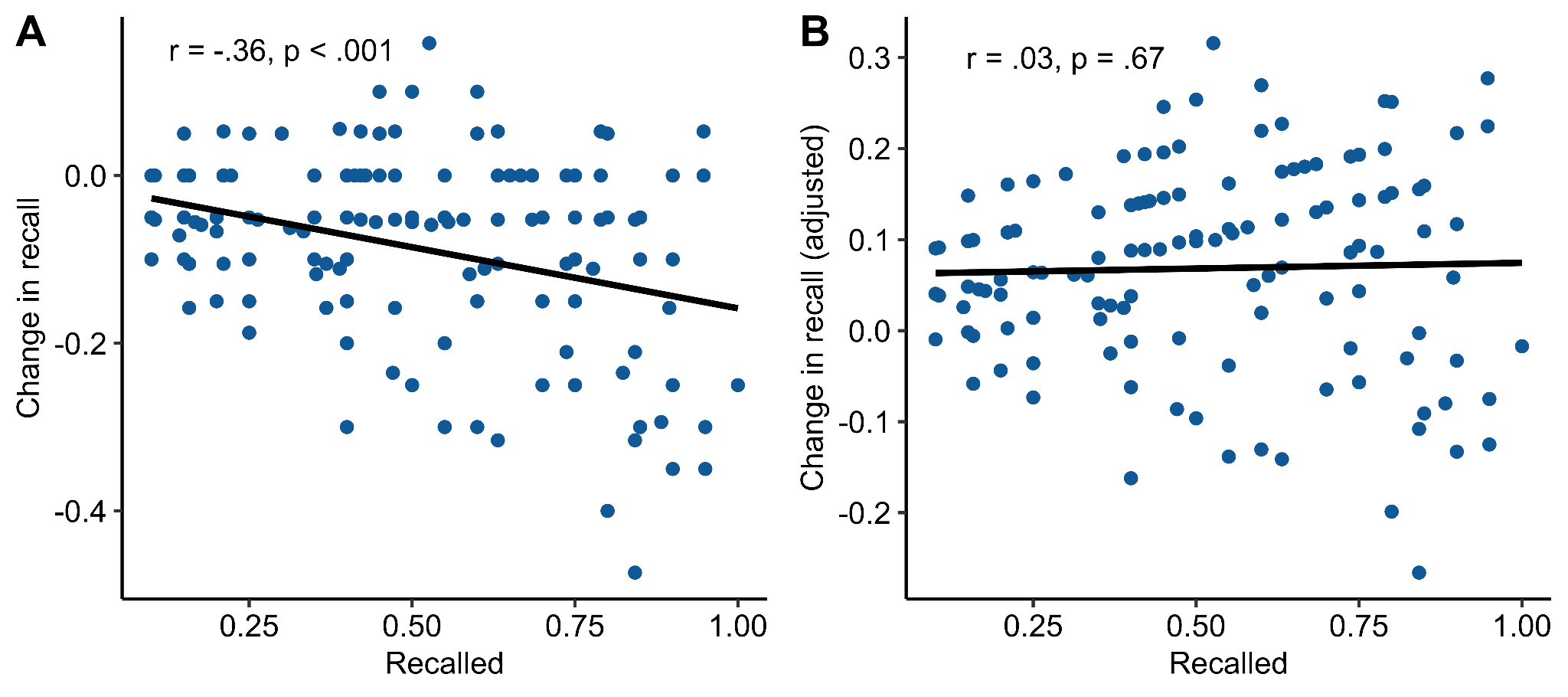


**Figure S1**. Adjusted change in recall score. **A** - Immediate recall accuracy significantly predicts the unadjusted change in recall score. **B** - Adjusted change in recall after regressing out immediate recall accuracy. This adjusted score is no longer associated with immediate performance.
